# Healthcare spending in the State of Louisiana

**DOI:** 10.1186/s12913-019-4275-y

**Published:** 2019-07-09

**Authors:** Blake P. Kruger, Jeremiah R. Brown

**Affiliations:** 1 Jacobs School of Medicine & Biomedical Sciences, State University of New York at Buffalo, Buffalo, NY USA; 2 Dartmouth College, Departments of Epidemiology, Biomedical Data Science, and The Dartmouth Institute for Health Policy and Clinical Practice, Geisel School of Medicine, Hanover, NH USA

**Keywords:** Healthcare spending, Hospital utilization, Outcomes research, Medicare

## Abstract

**Background:**

The State of Louisiana spends the most on Medicare beneficiaries per capita, but reports greater disparities in health status and death rates than other states. This project sought to investigate the associations between healthcare intensity, healthcare spending, and mortality in Louisiana.

**Methods:**

We used a 100% sample of 2014 Medicare claims data with beneficiaries assigned to hospital referral regions in Louisiana using small area analysis. We used simple and multivariable linear regression modelling to evaluate associations between healthcare intensity, healthcare spending rates, and mortality rates. We adjusted for age, sex, race, and population health risk factors.

**Results:**

We found no statistically significant associations between our measured variables when adjusted for age, sex, and race. These results were consistent after further adjusting mortality for population health risk factors.

**Conclusions:**

To our knowledge, no prior studies have investigated the associations between healthcare intensity, healthcare spending, and mortality in Louisiana. Our findings suggest that increased healthcare spending in Louisiana may not improve survival. Identifying more granular aspects of healthcare contributing to spending patterns in Louisiana may provide targets for future quality improvement work.

## Background

The United States spends more on healthcare services and reports lower life expectancy and higher infant mortality than other high-income nations [1]. In 2014, the United States spent $3.0 trillion on healthcare with costs projected to reach $5.7 trillion by 2026 [2, 3]. Drivers of increased spending include a growing population, an aging population, and uncontrolled service prices [4, 5]. Increasing healthcare spending diverts funding from social programs that may improve population health outcomes [1, 6]. As healthcare needs and costs have risen, so have per capita out-of-pocket medical expenditures [7–9]. In the United States, there is a strong association between the intensity of inpatient care and healthcare spending in the Medicare population [10–12], but the association between spending and mortality remains elusive and highly contentious [1, 10, 13–19].

The State of Louisiana spends the most on Medicare beneficiaries per capita, but reports greater disparities in health status and death rates than other states [11, 20]. With approximately 62.0% of citizens in Louisiana over the age of 65 enrolled in Medicare, the State of Louisiana spent over $4.5 billion on the Medicare program in 2014 [11, 21]. Despite an active national dialogue, we know of no prior studies that have investigated the associations between care intensity, spending, and outcomes in the State of Louisiana.

We investigated the associations between healthcare intensity, healthcare spending, and mortality in Louisianan Medicare beneficiaries. Understanding the relationship between healthcare intensity, healthcare spending and mortality among Medicare beneficiaries will help to facilitate further research into improving healthcare delivery in the State of Louisiana.

## Methods

This study (STUDY00030747) was approved by the Center for the Protection of Human Subjects; because data were deidentified, informed consent was waived.

### Cohort creation & data sources

We used two publicly-available data sources: the Dartmouth Atlas Project database and the Behavioral Risk Factor Surveillance System database [11, 22, 23]. The Dartmouth Atlas Project database provided 2014 Medicare claims data. We included all Medicare beneficiaries aged 65–99 years old that were enrolled in Medicare Parts A & B for at least one year. Using small-area analysis, we assigned included beneficiaries to 10 hospital referral regions in the State of Louisiana [10]. We found the prevalence of physical activity, obesity, and smoking for each hospital referral region in Louisiana by linking the Behavioral Risk Factor Surveillance System database with geographic boundaries provided by the Dartmouth Atlas Project. The Dartmouth Atlas Project and Behavioral Risk Factor Surveillance System databases were then merged without population density weights at the hospital-referral region level. National benchmarks were generated using patients that met the same criteria for all hospital referral regions across the United States.

### Primary outcomes

We had two primary outcomes of interest. We sought to assess the associations between: [1] healthcare intensity and healthcare spending and [2] healthcare spending and mortality. Our secondary outcome of interest was to assess the association between healthcare intensity and mortality.

We defined healthcare intensity to be synonymous with the hospital care intensity index. The hospital care intensity index is a standardized measure of inpatient care and was calculated for each hospital referral region by taking the ratio of the average number of inpatient days and the average number of physician visits compared to the national average that patients experienced over the last two years of life [10]. Calculating the hospital care intensity index over the last two years of life minimizes the impact of region-specific illness rates and illness trajectory [10]. These data can be found in the 2014 end-of-life chronic illness data file on the Dartmouth Atlas Project website [11].

We defined healthcare spending to be synonymous with Medicare Parts A & B spending. Spending rates were determined using all filed claims in 2014 and were indirectly adjusted for age, sex, and race. Price-adjustment was not used because price does not drive regional Medicare spending variation [24]. These data can be found in the 2014 Medicare spending data file on the Dartmouth Atlas Project website [11].

We defined mortality to be rates of death in 2014. These rates were also indirectly adjusted for age, sex, and race. Using a previously established mortality-adjustment methodology [25], we reduced observational intensity bias by 65% by correcting for major population health risk factors, namely obesity, physical activity and smoking [22, 23, 25]. These data can be found in the 2014 Medicare mortality file on the Dartmouth Atlas Project website [11].

### Statistical analyses

Associations between healthcare intensity, healthcare spending rates, and mortality rates were assessed using linear regression modelling at the hospital referral region-level. We used a simple linear regression model to assess for any association between healthcare intensity and healthcare spending rates. We used simple linear regression models to assess for any associations between healthcare spending rates and age, sex, and race-adjusted mortality rates, as well as healthcare intensity and age, sex, and race-adjusted mortality rates. We used multivariable linear regression models to reduce variance in mortality rates by adjusting for age, sex, race, and population health risk factors (smoking, physical inactivity, and obesity) [25]. The beta coefficient (ß) represents the relative strength of each independent variable in changing the dependent variable. The coefficient of determination (R^2^) represents the percent of each hypothesized phenomenon explained by the model. *P*-values that were less than or equal to 0.05 were interpreted as significant. We created turnip plots and maps to provide visual acuity in understanding fold variations for each major variable. The simple linear regression models in these analyses used adjusted and aggregated rates as: (Healthcare Spending) = ß (Healthcare Intensity) + c, with ‘Healthcare Spending’ exchanged for ‘Mortality,’ and ‘Healthcare Intensity’ exchanged for ‘Healthcare Spending’ as each hypothesis required. The ‘c’ term indicates the y-intercept generated with the model. Multivariable analyses expanded the dependent variable terms to include population health risk factors as each hypothesis required. Assumptions inherent in the regression models were not individually tested. We performed all analyses and plots using StataIC Version 15 (College Station, Texas) [26]. We generated all maps using Environmental Systems Research Institute ArcGIS software (Redlands, California) [27].

## Results

### Cohort characteristics

We identified a total of 423,391 Medicare beneficiaries in the State of Louisiana (Table 1). In 2014, compared to the national average, Louisiana provided more intense inpatient healthcare services (1.03 versus 1.00) and spent more on healthcare services ($10,094 per beneficiary versus $9589 per beneficiary). Furthermore, relative to the national average, Louisiana had a lower percentage of beneficiaries that were enrolled in health maintenance organizations (30% versus 32%) and over double the number of Black Medicare beneficiaries (21% versus 8%). More individuals in Louisiana were smokers, obese, and physically inactive. Finally, social security recipients in Louisiana received less per capita assistance ($1106 versus $1215) than the national average.

**Table 1:** Characteristics of Louisianan Medicare Beneficiaries. Characteristics of the Medicare beneficiary population at the national and Louisianan hospital referral region (HRR)-level derived from a 100% sample of 2014 Medicare Parts A & B claims [11], the 2010 United States Census [50], the 2014 Old-Age, Survivors, and Disability Insurance database [51], the 2014 Behavioral Risk Factor Surveillance System database [22], the 2010 Master Area Block Level Equivalency/Geocorr2k Geographic Correspondence Engine [52], and the 2014 Dartmouth Atlas Project Geographic Information System database file [11]

	National	LA HRRs^†^ *(n = 10)*
Average Hospital Care Intensity Index	1.00	1.03
Average Medicare Spending per Beneficiary^‡^ ($)	9589	10,094
Total Number of Beneficiaries (n)	29,586,354	423,391
Total Number of Beneficiary Deaths^§^ (n)	1,868,812	30,756
Total Population (n)	308,745,538	4,658,282
Demographics of Beneficiaries (%)		
Aged 75+	–	5.5%
Women	55%	56%
Black	8%	21%
Non-Black	92%	79%
Risk Factors (%)		
Smokers	20%	22%^*^
Physically Inactive	24%	31%^*^
Obese (> 30 body mass index)	26%	34%^*^
Beneficiaries Enrolled in Health Maintenance Organizations^•^ (%)	32%	30%
Social Security Status	National	State
Total Recipients (n)	59,077,158	854,211
Social Security Income per Beneficiary ($)	1215	1106

^**-**^Indicates no data was available

^**†**^Counties that crossed hospital referral region borders were silenced. Slidell was the only exception

^**§**^Age-, sex-, and race-adjustments were used for the calculation of total number of beneficiary deaths

^•^Age-, sex-, and race-adjustments were used for the calculation of percent of beneficiaries enrolled in health maintenance organizations

^*^Non-national Behavioral Risk Factor Surveillance System measures were allocated in an unweighted fashion using a Dartmouth Atlas Project merging file. West Feliciana Parish had no data available

^**‡**^Per capita spending is age, sex, and race-adjusted. Total Medicare spending was found using average reimbursements per enrollee multiplied by the number of enrollees to approximate total spending. Risk-bearing health maintenance organization enrollees were excluded

### Geographic variation

**Fig. 1:**
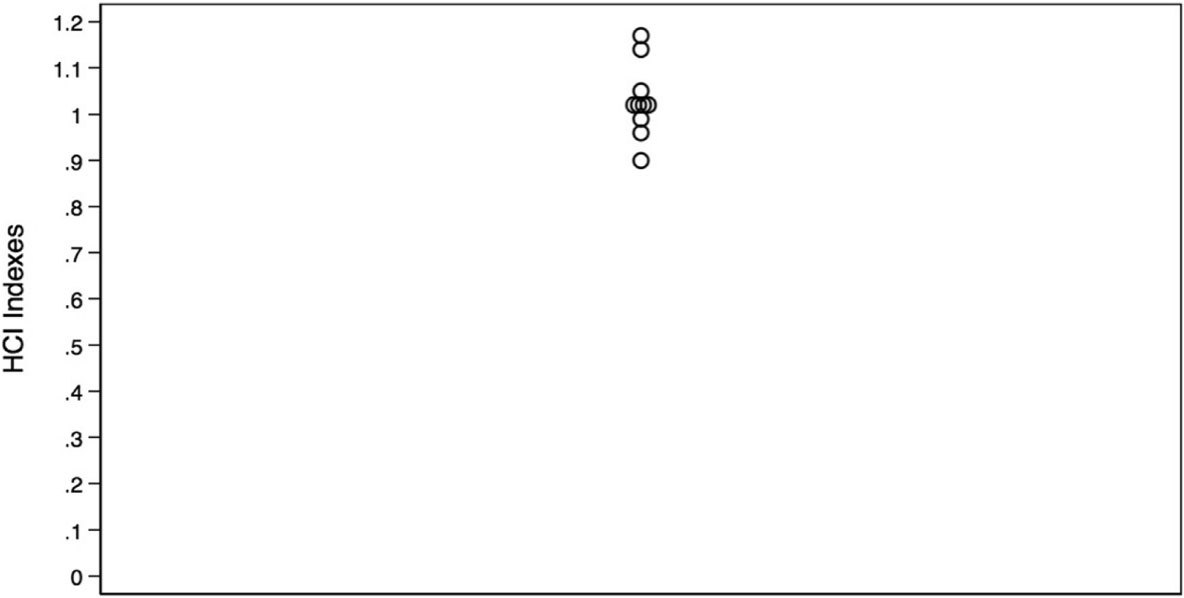
HRR-Level Hospital Care Intensity Indexes. Depicted are hospital care intensity (HCI) indexes by hospital referral region (HRR) in Louisiana *(Dartmouth Atlas Project, 2014)*

**Fig. 2:**
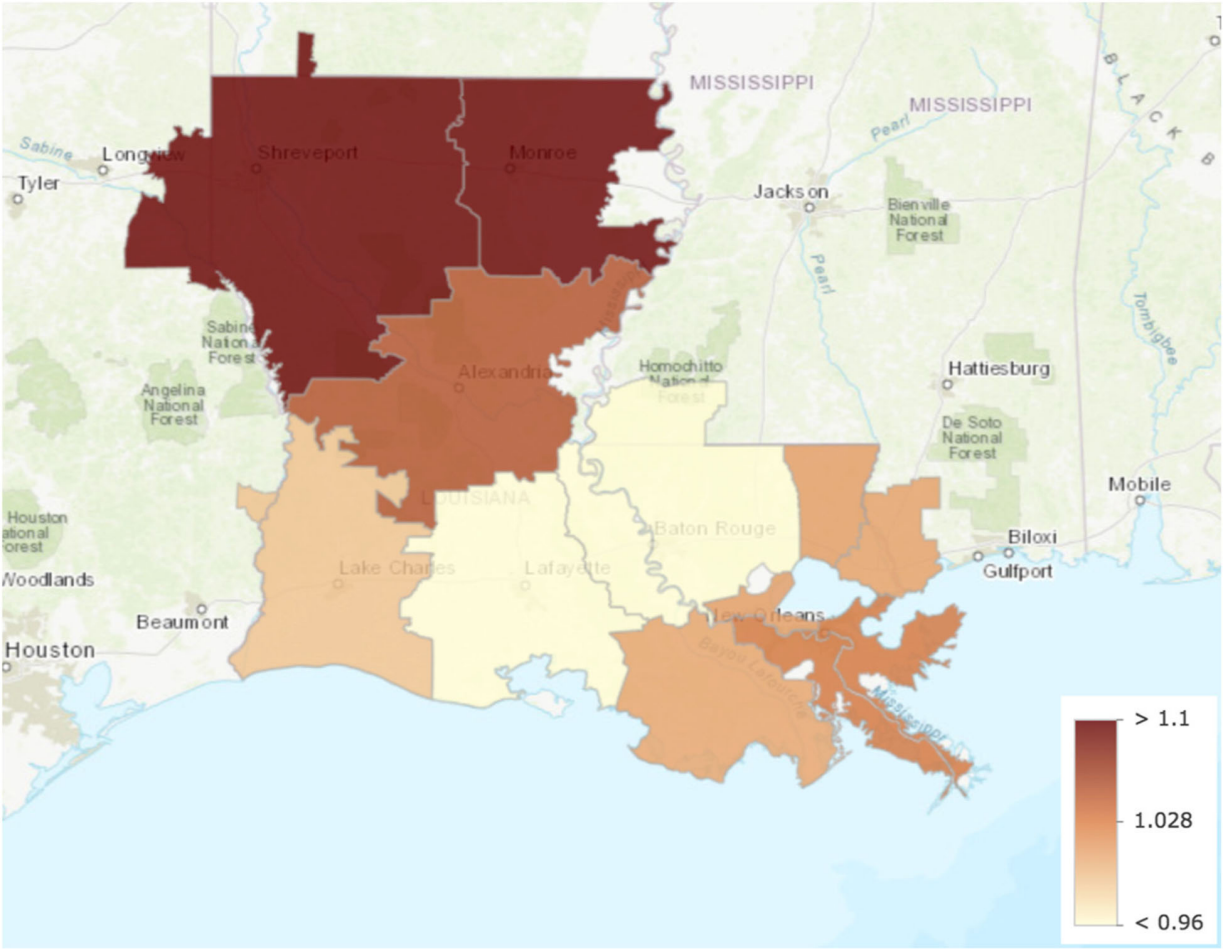
Geographic Depiction of HRR-Level Hospital Care Intensity Indexes. Depicted is the variation in hospital care intensity indexes by hospital referral region in Louisiana. *(Dartmouth Atlas Project, 2014)*

**Fig. 3:**
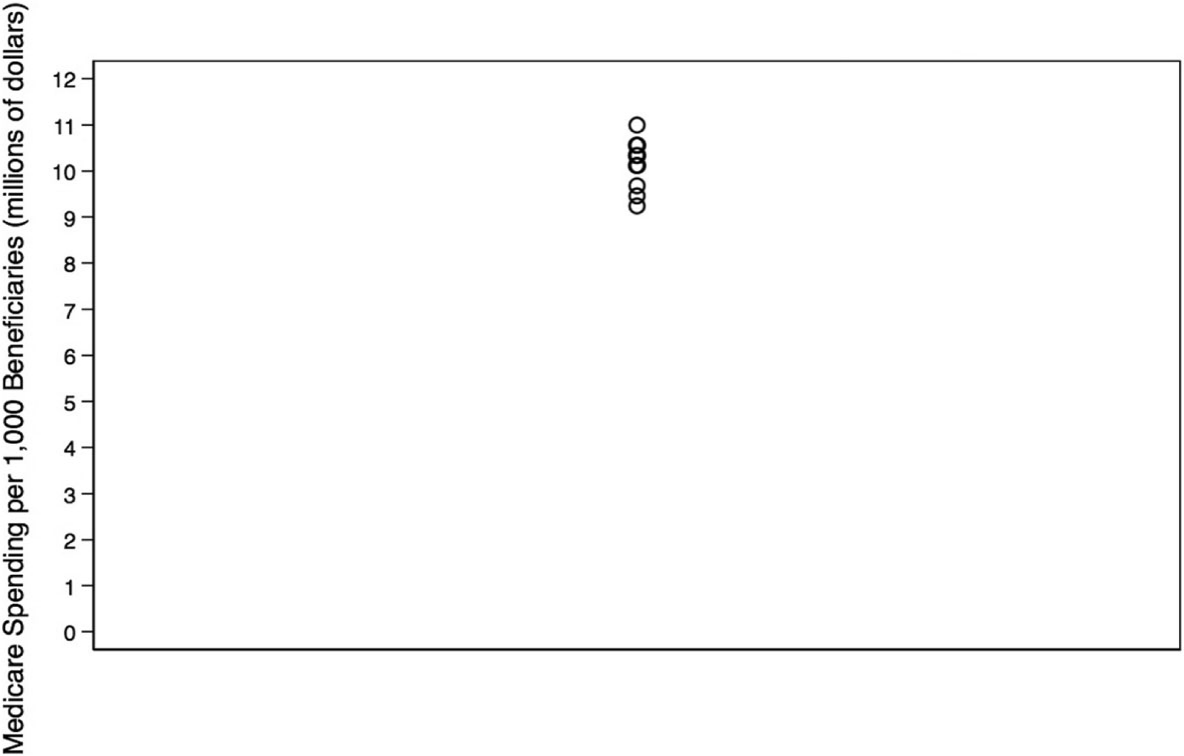
HRR-Level Total Medicare Parts A & B Spending. Depicted are Medicare Parts A & B spending rates per 1000 Medicare beneficiaries by hospital referral region in Louisiana *(Dartmouth Atlas Project, 2014)*

**Fig. 4:**
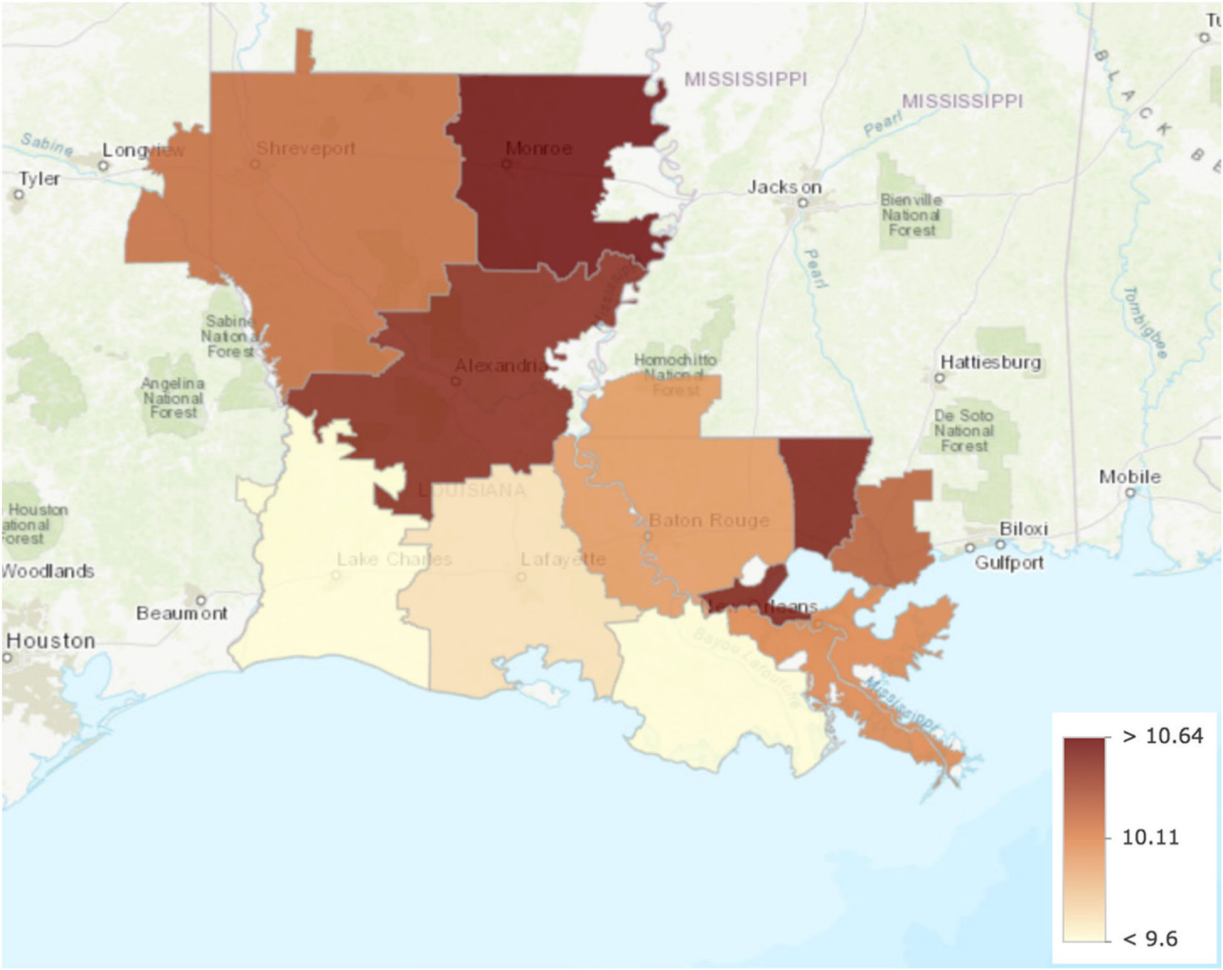
Geographic Depiction of HRR-Level Total Medicare Parts A & B Spending Rates. Depicted is the variation in Medicare Parts A & B spending rates per 1000 Medicare beneficiaries (in millions of dollars) by hospital referral region in Louisiana *(Dartmouth Atlas Project, 2014)*

**Fig. 5:**
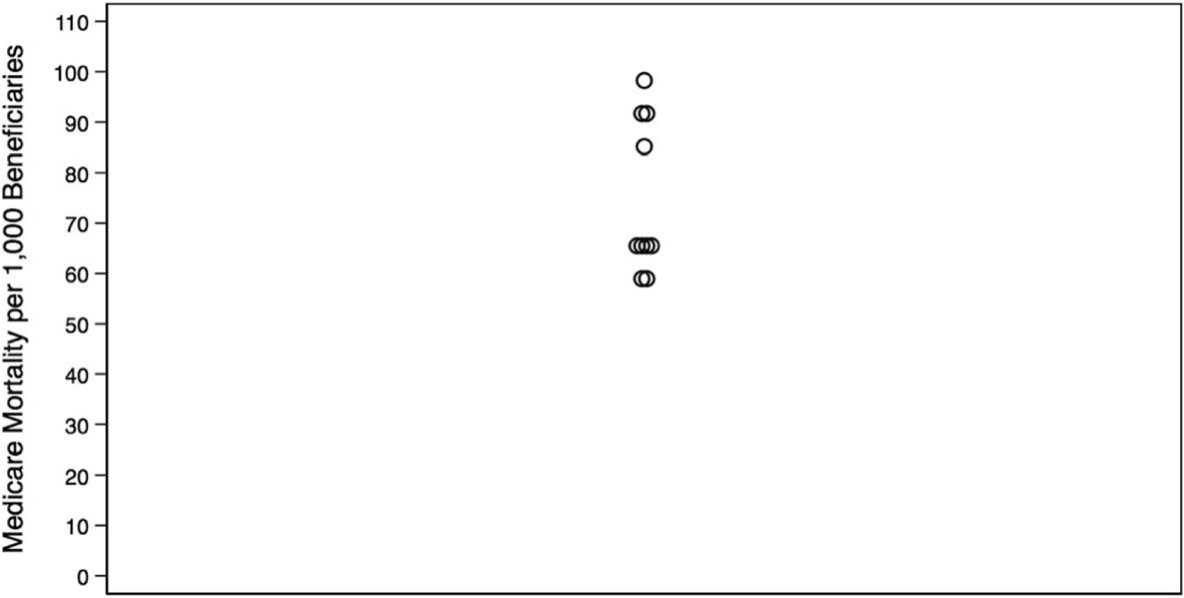
HRR-Level Medicare Mortality per 1,000 Beneficiaries. Depicted are Medicare mortality rates per 1000 Medicare beneficiaries by hospital referral region in Louisiana *(Dartmouth Atlas Project, 2014)*

**Fig. 6:**
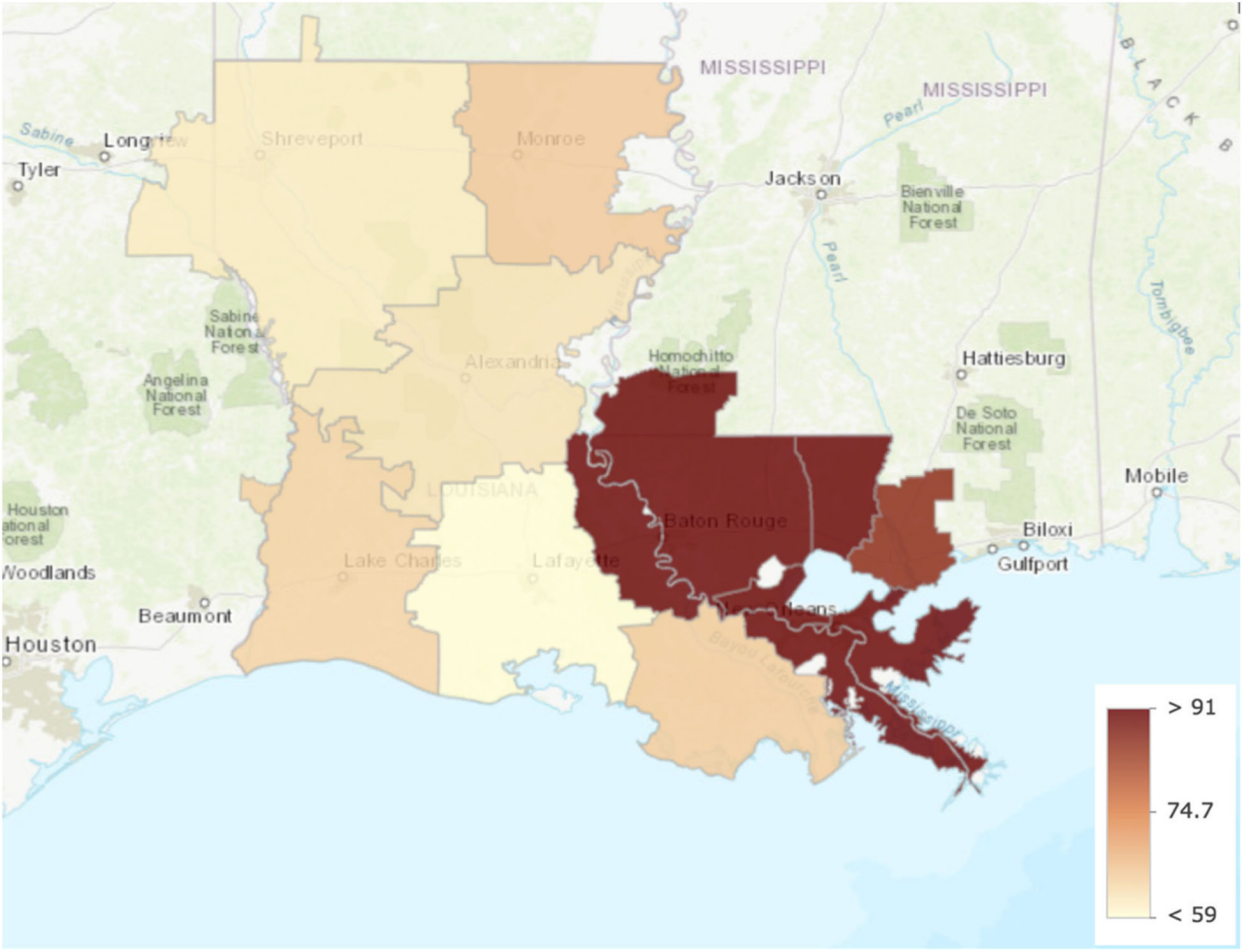
Geographic Depiction of HRR-Level Medicare Mortality Rates. Depicted is the variation in Medicare mortality rates per 1000 Medicare beneficiaries by hospital referral region in Louisiana *(Dartmouth Atlas Project, 2014)*

**Fig. 7:**
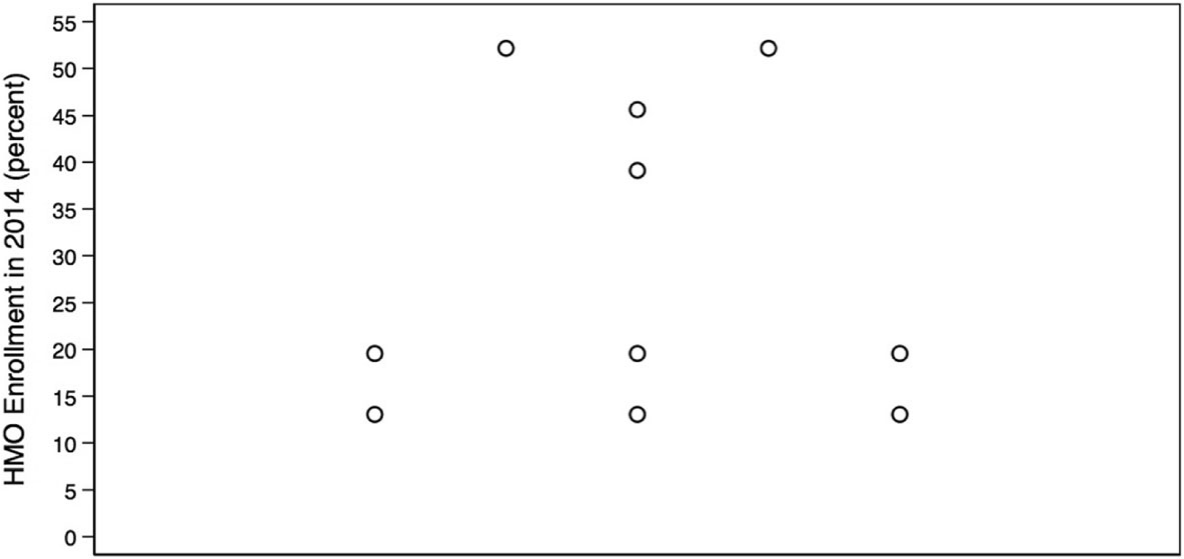
HRR-Level Percentages of HMO Enrollment. Depicted are percentages of health maintenance organization (HMO) enrollment by hospital referral region in Louisiana *(Dartmouth Atlas Project, 2014)*

**Fig. 8:**
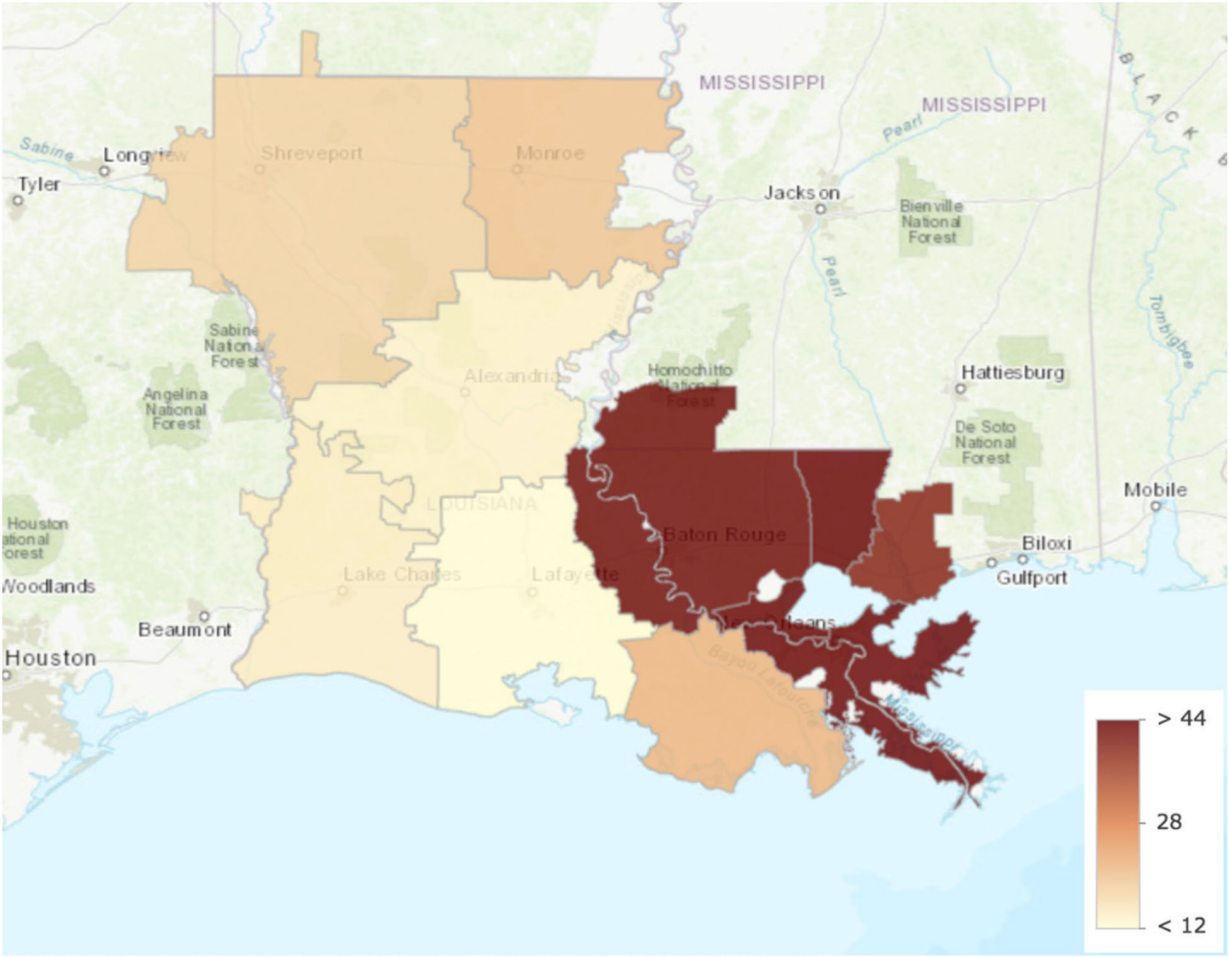
Geographic Depiction of HRR-Level HMO Enrollment Percentages. Depicted is the variation in percentages of health maintenance organization enrollment by hospital referral region in Louisiana *(Dartmouth Atlas Project, 2014)*

We identified Monroe as the only outlier with greater healthcare intensity than other hospital referral regions in Louisiana. Data from all other measures lay within the first quartile. Care intensity indexes (1.27, Figs. 1 and 2), spending rates (1.20, Figs. 3 and 4), and mortality rates (1.78, Figs. 5 and 6) showed seemingly large fold-variations. The fold-variation in health maintenance organization enrollment also seemed substantial (1.82, Figs. 7 and 8).

### Trends in healthcare services, spending, & mortality

**Fig. 9:**
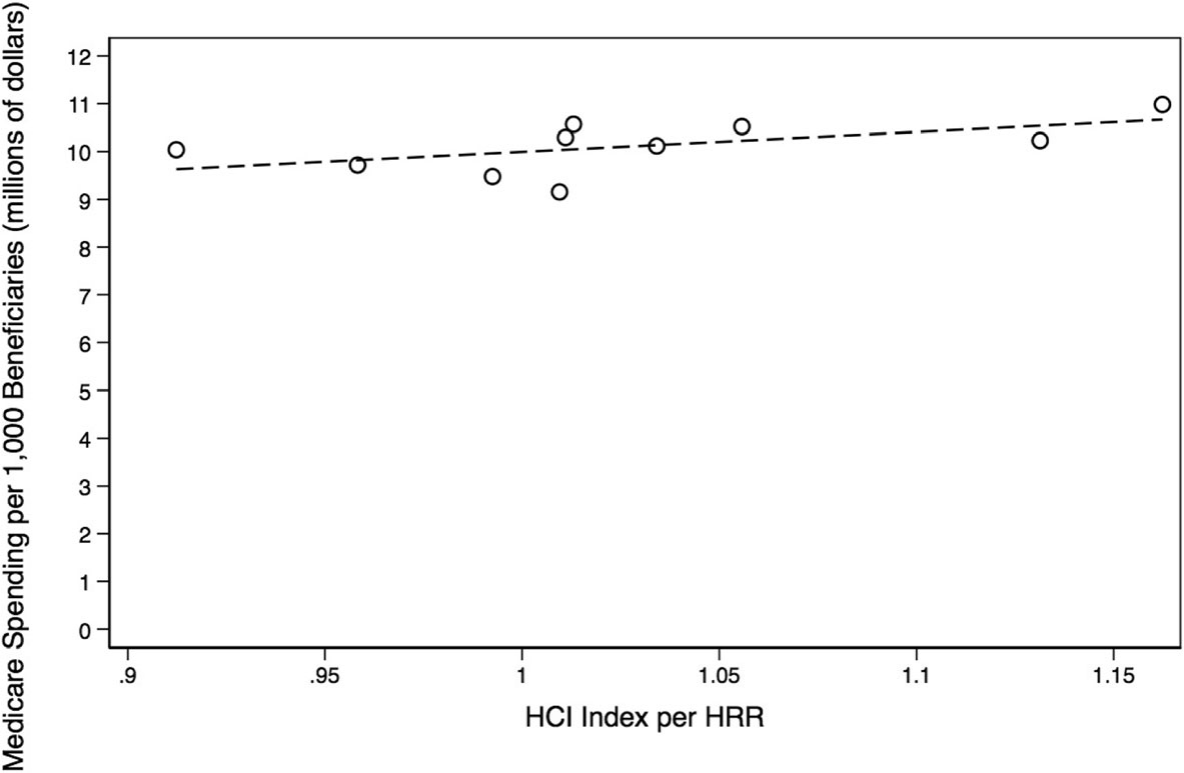
Total Medicare Spending versus Hospital Care Intensity Indexes. Depicted is a lack of association between hospital referral region-level care intensity indexes and Medicare Parts A & B spending per 1000 Louisianan Medicare beneficiaries. From left-to-right, the hospital referral regions with the two lowest care intensity indexes are Baton Rouge and Lafayette, respectively. From right-to-left, the referral regionss with the two highest care intensity indexes are Monroe and Shreveport, respectively *(Dartmouth Atlas Project, 2014)*

**Fig. 10:**
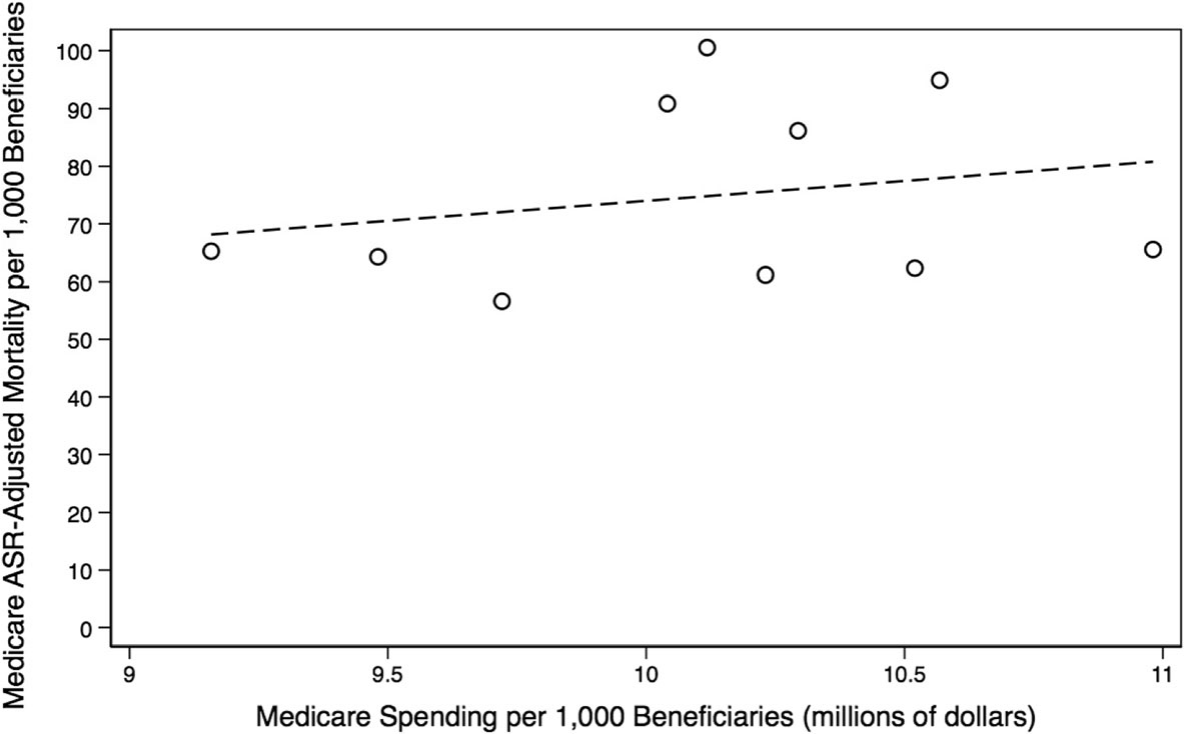
ASR-Adjusted Medicare Mortality versus Total Medicare Spending. Depicted is a lack of an association between hospital referral region-level Medicare Parts A & B spending per 1000 Louisianan Medicare beneficiaries and age, sex, and race (ASR)-adjusted Medicare mortality per 1000 Medicare beneficiaries. From left-to-right, the referral regions with the two lowest spending rates are Houma and Lake Charles, respectively. From right-to-left, the referral regions with the two highest spending rates are Monroe and Metairie, respectively *(Dartmouth Atlas Project, 2014)*

As shown in Table 2, we found no association between healthcare intensity and spending (ß = 4.13, *p* = 0.09; Fig. 9). We also observed no association between healthcare spending and age, sex, and race-adjusted mortality (ß = 6.94, *p* = 0.52; Fig. 10). These results were consistent when adjusting mortality for age, sex, race, and population health risk factors (ß_spending_ = 12.60, ß_smoking_ = 0.31, ß_inactivity_ = − 4.96, ß_obesity_ = 3.35, *p* = 0.74; Table 2). Comparing healthcare intensity to age, sex, and race-adjusted mortality also yielded no meaningful association (ß = -67.72, *p* = 0.39, Table 2). These results were consistent when adjusting mortality for age, sex, race, and population health risk factors (ß_care_ = − 22.64, ß_smoking_ = 0.55, ß_inactivity_ = − 2.24, ß_obesity_ = 0.62, *p* = 0.91; Table 2).

**Table 2:** Summary Table of Regression Models. A table presenting all analyses performed at the hospital referral region level with delineated age, sex, and race-adjustments, as well as age, sex, race, and population health-adjustments *(Dartmouth Atlas Project, 2014; Behavioral Risk Factor Surveillance System, 2014)*

Analyses	R^2^-Unadjusted	R^2^-Adjusted	*P*-Value	Inference
Primary Outcomes				
Care Intensity to Spending	0.2370	−0.3218	0.0872	There may be no association between care intensity and spending.
Spending to Age, Sex, Race-Adjusted Mortality	0.0527	−0.0657	0.5236	There may be no association between spending and mortality.
Spending to Age, Sex, Race, Population Health-Adjusted Mortality	0.2819	−0.2925	0.7449	There may be no association between spending and mortality.
Secondary Outcomes				
Care Intensity to Age, Sex, Race-Adjusted Mortality	0.0948	−0.0184	0.3868	There may be no association between care intensity and mortality.
Care Intensity to Age, Sex, Race, Population Health-Adjusted Mortality	0.1529	−0.5247	0.9129	There may be no association between care intensity and mortality.

## Discussion

To our knowledge, we are the first study to describe the associations between healthcare intensity, spending, and mortality rates among Louisianan Medicare beneficiaries. We found that no associations exist between healthcare intensity and spending, spending and mortality, as well as healthcare intensity and mortality. The lack of an association between healthcare intensity and spending may indicate that outpatient care in Louisiana is becoming more highly utilized and thus our measure of inpatient care intensity is becoming less sensitive [10, 28, 29]. These findings may also reflect that spending more on healthcare in Louisiana may not improve rates of survival and should prompt reflection as to the role social programs play in producing more auspicious health outcomes for Louisianans [1, 6]. Because we did not observe an association between care intensity and mortality, our findings may suggest that practice patterns differ across the State [16, 30–33]. Sustaining the current trajectory of national healthcare spending would have dire implications for the future viability of our healthcare system [7, 34, 35]. Louisiana has the opportunity to lead national healthcare reform by leveraging a unified approach to improving the cost-effectiveness of healthcare that is rooted in evidence-based medicine, shared decision-making practices, and the Model for Improvement [33, 36].

### Care Intensity & Spending in the state of Louisiana

**Fig. 11:**
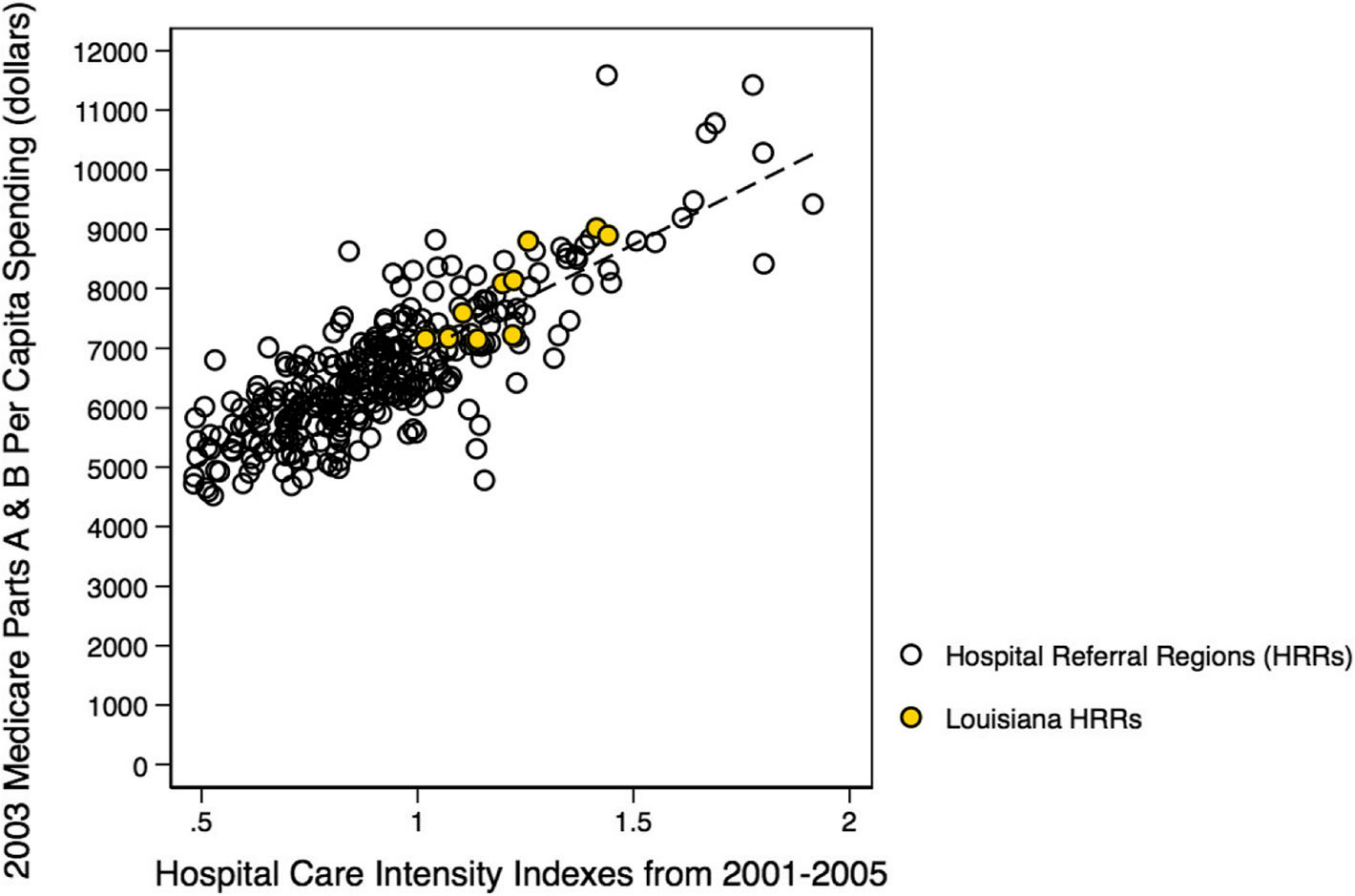
Replicated '*Tracking Medicine*' Analysis. Depicted is a reproduced national hospital referral region analysis from *Tracking Medicine*, with Louisianan referral regions highlighted, presenting the positive association between aggregated 2003 Medicare Parts A & B spending to care intensity constructed from hospital care intensity indexes aggregated from 2001 to 2005 using a decedent cohort over the last two years of life. These data are constructed using a 20% sample of Medicare beneficiaries for studied years *(Dartmouth Atlas Project)* [10]

**Fig. 12:**
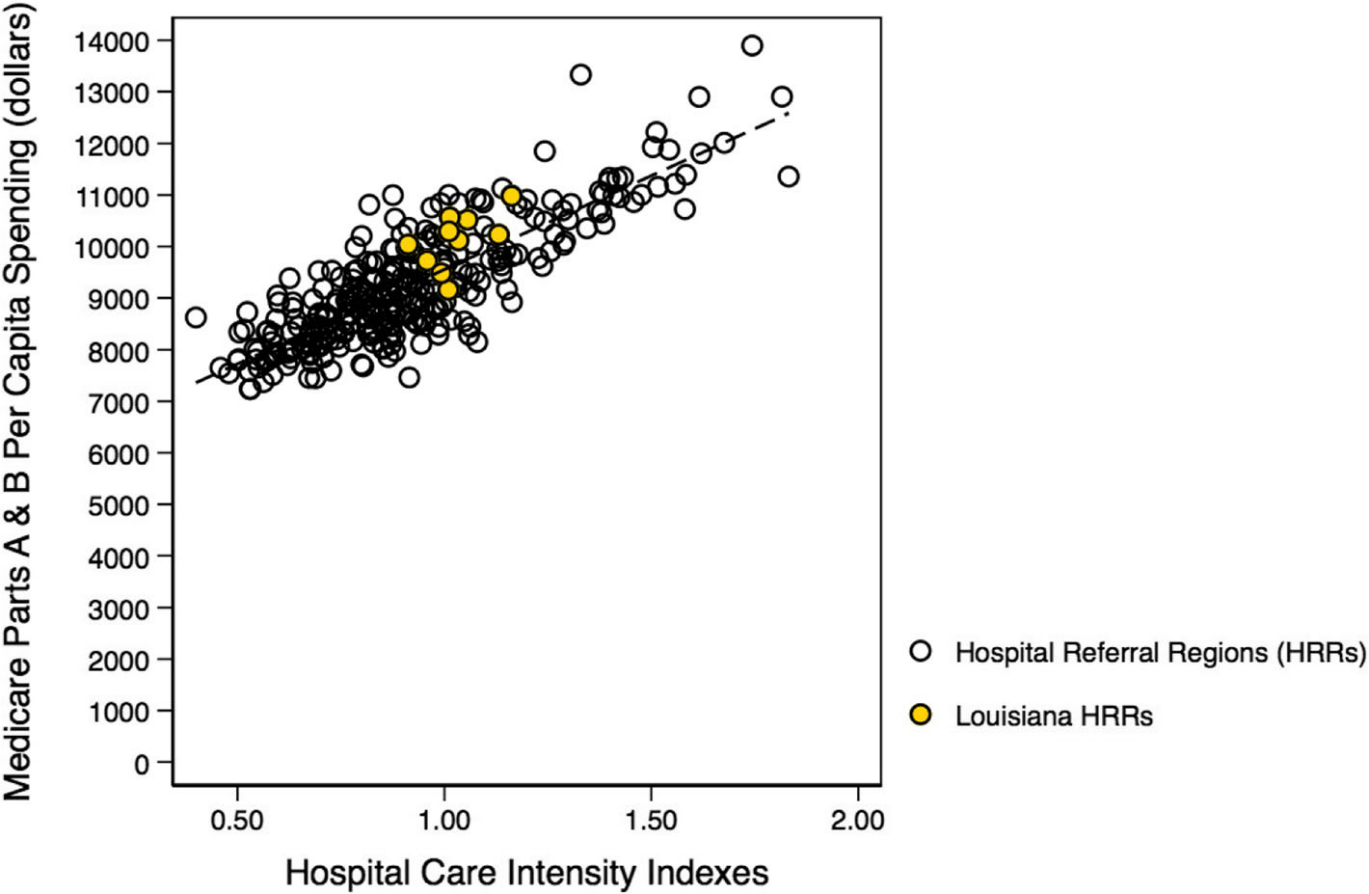
Updated '*Tracking Medicine*' Analysis. Depicted is an updated national hospital referral region analysis, presenting the positive association between Medicare Parts A & B spending to care intensity constructed from hospital care intensity indexes aggregated from using a decedent cohort over the last two years of life. These data are constructed using a 100% sample of Medicare beneficiaries during calendar year 2014 *(Dartmouth Atlas Project, 2014)*

Our findings, describing the role of care intensity and spending for the State of Louisiana, provide the first description of the Louisianan healthcare delivery system led by the Louisianan Alliance of Public-Private Partnership Hospitals after the 2013 restructuring of the Louisiana Charity Hospital System [37]. Although previous studies have identified strong, positive associations between inpatient care and Medicare spending on a national level [10, 16], this investigation does not support these prior findings for the State of Louisiana. We conducted a more detailed analysis comparing state-level to national-level care intensity and spending associations, and while the previously observed strong, positive association (ß = 3663.95, *p* < 0.01) still holds on a national-level (ß = 3648.69, *p* < 0.01) [10, 11], the last two decades have seen the significant, positive association (ß < 0.01, *p* < 0.01) become insignificant (ß = 0.02, *p* = 0.09) for hospital referral regions in Louisiana (Figs. 11 and 12) [11]. This shift may corroborate the inference that outpatient care in Louisiana is becoming more highly utilized and thus our measure of inpatient care intensity is now less sensitive than in prior studies [10, 28, 29]. It is not unreasonable to believe that healthcare delivery and spending patterns of Louisianan hospitals have changed with the passage of the Patient Protection and Affordable Care Act in 2010 and the restructuring of the Louisiana Charity Hospital System in 2013 [37–39].

These findings have direct implications as to how care can be improved in Louisiana. A recent publication has called for the reduction of healthcare employment growth [40], yet Louisiana currently has a modest number of health sector jobs per capita and will require an additional 392 primary care physicians by 2030 to maintain current primary care utilization rates [40, 41]. Another way to improve care and reduce costs may be to optimize the cost-effectiveness of treatment. The costs and outcomes of prostate cancer treatments have been well-studied [42–44]. In Louisianan Medicare beneficiaries, rates of treatment per 1000 male Medicare beneficiaries diagnosed with prostate cancer over the age of 75 exceed the national rate for radiation therapy (297.13 versus 258.38) and hormone therapy (470.57 versus 357.29), but not for delayed treatment (305.40 versus 339.09) [11, 45]. This may reflect over-utilization and prompt further investigation. Projects seeking to alter clinical practice should be constructed to respect patient autonomy, match clinical guidelines, and weigh individual risks and benefits accompanying tests and procedures [46–48].

### Spending and mortality in Louisiana

Prior analyses have been performed to assess the association between spending and mortality and have reached varied conclusions regarding what associations exist between spending and mortality [1, 10, 13–19]. As the scope of these reports diminish from national to condition-specific analyses this association generally shifts from no association to a positive association [10, 13–19]. For Louisiana, we observed no association between spending and mortality, adjusting mortality for age, sex, and race. Such results were consistent when adjusting mortality for age, sex, race, and population health risk factors that correct for variable illness rates across the population [25]. These findings have powerful policy implications and may suggest that meaningful systems-based innovations or quality improvement projects could simultaneously reduce healthcare costs and improve care quality [13, 33, 36]. For example, our findings may be used to prompt policymakers to reflect on investing in forward-thinking, prophylactic social service programs as Louisiana continues to address its chronic billion-dollar budget shortfall and changing healthcare landscape [1, 6, 37, 38, 49]. However, a better understanding of how spending and outcomes relate as the scope of these investigations decrease to the hospital-level are needed before meaningful changes can be implemented. Nevertheless, our findings may indicate that Medicare spending and survival are unrelated at the state-level.

Our work has limitations. One limitation to our methodology is that the geographic boundary file used to link county-level Behavioral Risk Factor Surveillance System data to the hospital referral region-level did not include population-density weights. Therefore, age, sex, race, and population health-adjusted mortality rates may overestimate the effect of risk behaviors when comparing a less densely populated to a more densely populated hospital referral region. Additionally, Behavioral Risk Factor Surveillance System data are taken from individuals from all ages and may not reflect the true prevalence of risk factors in the Medicare population. However, because of the agreement in findings when comparing our adjustment modalities, there is little reason to believe that this lack of weighting or age-specificity has significantly altered our conclusions. Another limitation includes the high percentage of beneficiaries enrolled in health maintenance organizations. With risk-bearing health maintenance organizations excluded from our spending rate generation, it is possible that the inclusion of these excluded Medicare beneficiaries may alter our findings (Range: 10.6 to 51.4%; Fig. 7). However, this limitation may have a lesser impact on our findings as Louisiana has lower health maintenance organization penetration when compared to the nation overall (Table 1). A final limitation is the use of ten data points for a multivariable regression, which may limit the reliability of findings irrespective of the number of beneficiaries that comprise the summative rates. Unfortunately, this is an unavoidable limitation as Louisiana has only ten hospital referral regions.

Directions for future investigations are numerous. Future analyses can improve on this work by using all-payer claims data and conducting these analyses on the patient- and hospital-level in Louisiana. Greater clarity surrounding these hospital-level findings are needed before further recommendations can be made. Other investigations may assess patterns of clinical practice and healthcare workforce distributions in Louisianan hospitals. Healthcare economists and providers can also work together to develop systems-level changes in clinical practice, better quality metrics, and provide guidance as to what are truly ‘meaningful’ fold-variations in care intensity, spending, and mortality. These changes could be modelled after studying how a low-spending, moderate care intensity, and low-mortality hospital referral region, like Houma, provides care.

## Conclusions

We found no associations between healthcare intensity, healthcare spending, and mortality for Louisianan Medicare beneficiaries. These findings could reflect that spending more on healthcare in Louisiana may not improve survival. Understanding these trends on the hospital-level and investigating clinical practice patterns can inform the development of lower-cost and higher-quality health systems for the State of Louisiana. Identifying more granular aspects of care that contribute to these spending patterns may provide targets for future quality improvement work.

## Data Availability

We used two publicly-available data sources: the Dartmouth Atlas Project database and the Behavioral Risk Factor Surveillance System database [11, 22, 23]. The Dartmouth Atlas Project database provided 2014 Medicare claims data and geographic boundaries for analysis (https://atlasdata.dartmouth.edu/static/research_data_archive). The Behavioral Risk Factor Surveillance System database provided adjustment measures (https://www.cdc.gov/brfss/annual_data/annual_2014.html).

## Abbreviations

ASR: Age, Sex, Race
HCI: Hospital Care Intensity
HMO: Health Maintenance Organization
HRR: Hospital Referral Region
